# Meaning maps capture the density of local semantic features in scenes: A reply to Pedziwiatr, Kümmerer, Wallis, Bethge & Teufel (2021)

**DOI:** 10.1016/j.cognition.2021.104742

**Published:** 2021-04-21

**Authors:** John M. Henderson, Taylor R. Hayes, Candace E. Peacock, Gwendolyn Rehrig

**Affiliations:** a Center for Mind and Brain, University of California, Davis, USA; b Department of Psychology, University of California, Davis, USA

**Keywords:** Eye movements, Scene perception, Attention

## Abstract

Pedziwiatr, Kümmerer, Wallis, Bethge, & Teufel (2021) contend that Meaning Maps do not represent the spatial distribution of semantic features in scenes. We argue that Pesziwiatr et al. provide neither logical nor empirical support for that claim, and we conclude that Meaning Maps do what they were designed to do: represent the spatial distribution of meaning in scenes.

Pedziwiatr et al. (2021, henceforth PKWBT) claim that Meaning Maps (Henderson & Hayes, 2017) “are insensitive to image meaning when predicting human fixations”. They reach this conclusion based on two problematic arguments. These arguments are: (1) Because Meaning Maps do not capture object-scene semantic consistency, they do not capture any aspects of semantic content; (2) Because Meaning Maps do no better than DeepGaze 2 (DG2) both generally and with regard to object-scene consistency, Meaning Maps reduce to the same type of non-semantic physical features used by DG2, a deep-learning saliency model trained on human eye movements (Kümmerer, Wallis, & Bethge, 2016). Neither of these conclusions follow from the premises, arguments, or data presented by PKWBT, and neither is correct.

Because PKWBT challenge the fundamental ability of Meaning Maps to represent meaning and their usefulness as a tool for studying meaning in scenes, we begin by touching on a few general preliminaries concerning Meaning Maps and the theory they were developed to investigate. This will then set the stage for our main points concerning PKWBT that follow.

First, the theoretical claim at stake in our work is simple: When humans visually perceive the real world, visual-spatial attention is driven in large part by our understanding and interpretation of what we are seeing, along with what we are trying to accomplish (Henderson, 2003, 2007, 2011). Research supporting this idea has a long history in visual cognition (Buswell, 1936; Yarbus, 1967) and is reinforced by a large body of evidence (Einhäuser, Rutishauser, ¨ & Koch, 2008; Tatler, Hayhoe, Land, & Ballard, 2011; Torralba, Oliva, Castelhano, & Henderson, 2006; Williams & Castelhano, 2019; Wu, Wick, & Pomplun, 2014). PKWBT acknowledge this point to some degree, allowing that there are task effects on attention in scenes while denying a general influence of semantic content. Importantly there is a growing body of behavioral and neural evidence that fundamental visual-spatial attention systems are strongly influenced by the semantic properties of meaningful visual stimuli, and indeed that semantic properties often override physical properties in the control of attention. This evidence has been observed in traditional attention paradigms (Malcolm, Rattinger, & Shomstein, 2016; Shomstein, Malcolm, & Nah, 2019), simplified object displays (Nuthmann, de Groot, Huettig, & Olivers, 2019), and in scene perception research (Võ, Boettcher, & Draschkow, 2019; Williams & Castelhano, 2019; Wu et al., 2014). At this point there is little remaining doubt that the influence of physical (image-based, non-semantic) saliency on visual-spatial attention can be overridden by many factors including meaning, and what remains to be determined is how this overriding is accomplished (Luck, Gaspelin, Folk, Remington, & Theeuwes, 2020).

Given the strong evidence for a central role of semantics in attentional control, why is there still such an emphasis on physical features in much of the attention literature, including the scene attention literature? We suspect that this focus on physical salience arises for at least two important reasons (Henderson, 2017). First, visual-spatial attention has traditionally been studied using stimuli that mostly or entirely lack semantic content, in large part because psychophysical and related methods are more easily deployed for such stimuli. Second, attentional modeling has similarly traditionally focused on physical features that exclude semantic content because modeling attention over image computable physical features has historically been more tractable than modeling over semantic features (though see Hayes and Henderson (2021)). Certainly, much has been learned about the fundamental nature of the attention system by taking this approach. At the same time, it is clearly not the whole story.

In visual cognition and cognitive neuroscience, a great deal of the evidence for the role of semantics on attention in complex real-world scenes has been based on manipulations of object-scene semantic relationships. For example, it is common to swap semantically consistent objects across scenes (e.g., classically swapping an octopus and a tractor in an underwater and farm scene respectively) to create semantic inconsistencies (Loftus & Mackworth, 1978). This is a powerful manipulation for investigating and establishing the causal role of semantics on attention, and it is one that we have often taken ourselves (Brockmole & Henderson, 2008; Henderson, Weeks Jr., & Hollingworth, 1999; Võ & Henderson, 2009). It is this approach that PKWBT also focus on. But a drawback of this method is that only one discrete region of a scene (the manipulated object) can be investigated in each scene, limiting the amount of data that can be collected and the conclusions that can be drawn about changes in semantic density across the entire scene.

In comparison to the literature on object-scene semantic consistency, in the literature investigating the role of physical salience on attention in scenes, physical properties are typically represented as a spatially continuous distribution of salience values across a scene in the form of a saliency map. The lack of an analogous spatially continuous representation of semantic properties has made it difficult to directly compare physical features to semantic features in scenes. Given these considerations, our goal in developing Meaning Maps was to generate a continuous representation of the spatial distribution of semantic features across a real-world scene in the same format as physical saliency maps, so that the two can be directly compared.

Because there have been no computational methods that can automatically produce semantic density maps, we capitalized on the semantic systems of human raters to tell us how meaning varies across scenes. Importantly, given that models based on physical salience do not consider global scene characteristics, in our initial work we purposefully focused on creating Meaning Maps that represented the distribution of context-free semantic density for local scene regions without taking context into account, in what we have called “context-free” Meaning Maps (Henderson, 2020; Henderson, Hayes, Peacock, & Rehrig, 2019). In other words, we purposefully excluded contextualized meaning such as object-scene semantic relationships from Meaning Maps, not because context would be impossible to include, but as a conscious decision given our specific goals at the time. Indeed, this is one of the reasons we created Meaning Maps instead of using rating paradigms that already existed (Antes, 1974; Mackworth & Morandi, 1967; t’ Hart, Schmidt, Roth, & Einhäuser, 2013).

Returning to PKWBT, the conclusion that Meaning Maps represent physical features rather than semantic features requires assuming that raters ignore the instructions they are given. In our original context-free rating procedure, subjects are asked to rate each individually-presented patch based on their assessment of how informative and recognizable that patch is. There is no reason to believe that raters perversely ignore these instructions and rate physical features instead. Indeed, we have successfully generated Meaning Maps designed to capture different semantic features simply by changing the rating instructions. For example, we have generated what we call “contextualized” Meaning Maps by presenting exactly the same patches used in context-free Meaning Maps, but with each individual patch shown with its scene (Peacock, Hayes, & Henderson, 2019). We have also generated “Grasp Maps” using exactly the same patches with instructions focused on whether the region depicts an entity that can be grasped (Rehrig, Peacock, Hayes, Henderson, & Ferreira, 2020). Importantly, when the instructions are changed, subjects change their ratings to reflect the semantic features they are asked to rate, leading to different maps, even though the physical features are held constant. If raters were simply rating physical features in each patch, the ratings would not change as a function of the semantic characteristics of the rating task. Therefore, contrary to the unsupported claim in the target article, we have direct evidence that Meaning Maps reflect semantic features. Furthermore, neurocognitive work in our lab provides converging evidence supporting this conclusion. For example, cortical areas along the ventral visual stream supporting higher level scene processing show activation that is associated with the semantic values of fixated scene regions captured by Meaning Maps, but not with simple visual features (Henderson, Goold, Choi, & Hayes, 2020). Ongoing research in our lab shows that this activation is more extensive than the activation associated with DG2, strongly suggesting that the two representations do not capture the same information. We also see a similar dissociation between physical salience and semantic features represented by Meaning Maps in EEG work examining the time-course of scene perception (Kiat, Hayes, Henderson, & Luck, 2020).

Meaning Maps offer a powerful general method for investigating the spatial distribution of semantic features (or what we have called “semantic density”) in complex scenes. By design, our original context-free Meaning Maps capture some aspects of scene meaning and not others. Contrary to the conclusion of PKWBT, this limitation in scope is not evidence that context-free Meaning Maps do not represent any aspect of meaning. PKWBT focus on object-scene semantic relationships, arguing that because neither context-free Meaning Maps nor DG2 account for the influence of object-scene consistency on attention, Meaning Maps and DG2 must reduce to the same type of non-semantic underlying representation. This conclusion obviously does not follow: The fact that two representational systems are equally unable to account for some phenomenon does not logically require the conclusion that they are identical to each other. Here the two types of representations fail for different reasons, as is often the case when competing models are compared.

There are many other aspects of scene meaning that the original context-free Meaning Maps also do not capture. For example, they do not capture aspects of meaning that depend on the viewer’s task. They do not capture the increased meaning of water to a thirsty person. They do not capture the heightened meaning of a spider to an arachnophobe. They do not capture changes to meaning that might arise as a consequence of an unfolding event. They do not capture individual differences in meaning based on the unique histories of individual people. They do not capture a viewer’s transient motivation. There is nothing about these cases that leads to the conclusion that Meaning Maps therefore do not represent scene meaning at all. And critically, it is a simple matter to extend the “classic” context-free Meaning Map approach to include additional types of semantic features, as we have done (Peacock et al., 2019; Rehrig et al., 2020). Importantly, whereas Meaning Maps can easily be extended to investigate a variety of semantic features, it is far less clear whether deep learning models like DG2 can ever in principle capture object-scene semantic features, or indeed any type of semantic feature.

PKWBT do a disservice to many fields of inquiry by incorrectly and unnecessarily dismissing a method that has already proven useful for investigating scene semantics across several domains of research, and that will likely continue to prove useful in new domains. For example, Meaning Maps have already been used to investigate scene memory (Bainbridge, Hall, & Baker, 2019; Ramey, Yonelinas, & Henderson, 2020), language production (Ferreira & Rehrig, 2019; Henderson, Hayes, Rehrig, & Ferreira, 2018; Rehrig et al., 2020), mind wandering (Krasich, Huffman, Faber, & Brockmole, 2020), active vision in immersive VR environments (Haskins, Mentch, Botch, & Robertson, 2020), and infant attentional development (Klotz, Hayes, Pomaranski, Henderson, & Oakes, 2021). Ongoing work in our lab uses Meaning Maps to investigate the nature and time-course of scene perception in the brain (Henderson et al., 2020; Kiat et al., 2020). These disparate lines of research demonstrate the utility of meaning maps for studying scene semantics. Questioning all of this work without basis unnecessarily confuses and undermines both existing and potentially new and important empirical and theoretical lines of investigation.

PKWBT also conclude that Meaning Maps do not predict attention in scenes as well as DG2 in a free viewing task. This conclusion is based on empirical results presented by PKWBT that will require replication and corroboration conducted in the course of normal science. Because it is not directly relevant to our main focus here, we set it aside for the purposes of this commentary.

In sum, there is neither empirical nor logical reason to dismiss Meaning Maps, and much reason to conclude that they do what they were designed to do: represent the spatial distribution of meaning in scenes.
